# Multi-Strategy Enhancement of YOLOv8n Monitoring Method for Personnel and Vehicles in Mine Air Door Scenarios

**DOI:** 10.3390/s25103128

**Published:** 2025-05-15

**Authors:** Lei Zhang, Hongjing Tao, Zhipeng Sun, Weixun Yi

**Affiliations:** School of Coal Engineering, Shanxi Datong University, Datong 037000, China; dtblack84@cumt.edu.cn (L.Z.); zhipengsun@sxdtdx.edu.cn (Z.S.); 230857002147@sxdtdx.edu.cn (W.Y.)

**Keywords:** YOLOv8n, PConv, mine air door, object detection

## Abstract

The mine air door is the primary facility for regulating airflow and controlling the passage of personnel and vehicles. Intelligent monitoring of personnel and vehicles within the mine air door system is a crucial measure to ensure the safety of mine operations. To address the issues of slow speed and low efficiency associated with traditional detection methods in mine air door scenarios, this study proposes a CGSW-YOLO man-vehicle monitoring model based on YOLOv8n. Firstly, the Faster Block module, which incorporates partial convolution (PConv), is integrated with the C2f module of the backbone network. This combination aims to minimize redundant calculations during the convolution process and expedite the model’s aggregation of multi-scale information. Secondly, standard convolution is replaced with GhostConv in the backbone network to further reduce the number of model parameters. Additionally, the Slim-neck module is integrated into the neck feature fusion network to enhance the information fusion capability of various feature maps while maintaining detection accuracy. Finally, WIoUv3 is utilized as the loss function, and a dynamic non-monotonic focusing mechanism is implemented to adjust the quality of the anchor frame dynamically. The experimental results indicate that the CGSW-YOLO model exhibits strong performance in monitoring man-vehicle interactions in mine air door scenarios. The Precision (P), Recall (R), and the map@0.5 are recorded at 88.2%, 93.9%, and 98.0%, respectively, representing improvements of 0.2%, 1.5%, and 1.7% over the original model. The Frames Per Second (FPS) has increased to 135.14 f·s^−1^, reflecting a rise of 35.14%. Additionally, the parameters, the floating point operations per second (FLOPS), and model size are 2.36 M, 6.2 G, and 5.0 MB, respectively. These values indicate reductions of 21.6%, 23.5%, and 20.6% compared to the original model. Through the verification of on-site surveillance video, the CGSW-YOLO model demonstrates its effectiveness in monitoring both individuals and vehicles in scenarios involving mine air doors.

## 1. Introduction

The coal mine air door is typically installed in vehicle transportation and pedestrian roadways, serving the dual purpose of regulating the passage of individuals and vehicles while also managing airflow [1]. Currently, some coal mines continue to employ manual or semi-manual methods for controlling these air doors. This approach not only results in poor timeliness and low operational efficiency but also increases the risk of unsafe incidents. Consequently, it is essential to investigate real-time monitoring systems for mine dampers, as this research holds significant implications for advancing intelligent construction practices and ensuring safe production within the coal mining industry.

In the traditional air door scenario, the monitoring method involves real-time oversight by a dispatcher who observes the conditions at both the inlet and outlet of the air door. Subsequently, the dispatcher manually operates the air door or utilizes various sensors to facilitate this process [2]. However, these methods are associated with several drawbacks, including high costs, low monitoring efficiency, and slow closure times for the air door. With advancements in artificial intelligence technology, object detection techniques based on deep learning have found extensive applications in previously underutilized scenarios within underground environments. These applications include areas such as miner safety equipment (e.g., helmets), monkey cars, coal gangue management, and other related fields [3,4,5,6]. Cui Lizhen et al. [7] employed a Convolutional Neural Network (CNN) model for feature extraction, considering the various movement states of underground personnel. They classified the behavior patterns of these individuals using a CNN-LSTM model. This model significantly improves accuracy and resilience against interference in personnel positioning within complex environments. However, its effectiveness depends on the quality of data provided by the sensors, and its adaptability to other devices is currently limited. Xiao Zhenjiu et al. [8] proposed a helmet detection algorithm based on YOLOv8n, which effectively monitored small target objects, such as helmets, by integrating modifications to the feature fusion network, feature pyramid, and partial convolution techniques. The model effectively adapts to the complex environment beneath the mine while maintaining a low computational cost. However, there is a slight increase in model complexity and the number of parameters compared to the original version. Furthermore, there is still potential for optimization in monitoring small targets and occluded objects. Xie Beijing et al. [9] developed a monkey car detection algorithm based on YOLOv8n, taking into account the varying human states of the monkey cars. The approach incorporates several innovative strategies: an adaptive direct square strategy is introduced to enhance image quality; a variable convolution technique is employed to improve the target receptive field within the C2f module; and a coordinate attention mechanism is integrated to effectively capture global key information. Consequently, this method significantly improves the detection accuracy of monkey cars. It enhances detection accuracy in complex backgrounds, such as dimly lit scenes and occlusions; however, there is still potential for further improvement in detection speed. Qin Yulong et al. [10] addressed the transportation challenges associated with large lump coal on belt conveyors by proposing a detection method based on YOLOv5s. This approach involves replacing specific standard convolution layers in the backbone network with parallel cavity convolutions and incorporating a joint attention module, thereby enhancing the monitoring capabilities for large lump coal. At the same time, the frequency of missed and incorrect detections of large lump coal has decreased. However, the computational complexity of the model has increased, and its adaptability in extreme scenarios requires further validation. Chenxi Liu et al. [11] proposed an object detection algorithm based on YOLOv8n. By incorporating the Multi-Head Self-Attention (MHSA) mechanism and modifying the loss function, the model significantly enhanced its ability to detect multiple objects while also improving positioning accuracy.

Currently, there is a limited amount of research focused on the intelligent recognition of air door scenarios. Therefore, it is essential to conduct research and apply target detection technology within the context of air door scenarios in coal mines.

For the monitoring task associated with air door scenarios, the primary detection targets to consider are vehicles and personnel passing through the air door. In these scenarios, the vehicle in question is an explosion-proof trackless rubber-wheeled vehicle. To further regulate the types of vehicles traversing the air door and to ensure the safety of mining operations, this paper identifies the specific type of explosion-proof trackless rubber-wheeled vehicle involved. License plate recognition is primarily employed for managing entry and exit in conventional intelligent vehicles [12]. However, this approach demonstrates low detection efficiency in complex mining environments, which are characterized by factors such as dim lighting conditions, obscured monitoring lenses due to construction activities, and coal dust obstructing license plates. Zhu Jinrong et al. [13] employed morphological operations in conjunction with Fourier descriptors to extract vehicle contour curves, subsequently facilitating vehicle recognition. This method is well-suited for high-speed traffic environments and can significantly reduce both false positives and missed detections. However, the model’s adaptability to complex traffic scenarios remains limited. To tackle the challenges associated with the hybrid detection and recognition of both large and small targets, Yu Jie et al. [14] proposed a vehicle recognition algorithm based on a cascade multi-task deep neural network. This approach integrates an enhanced YOLO algorithm with the DeepSort algorithm to achieve effective vehicle recognition, localization, and target tracking. The system is well-suited for complex environments and demonstrates robust performance in scenarios involving license plate ambiguity and occlusion. However, its tracking accuracy is vulnerable to the challenges presented by occlusion. To address the challenges posed by complex traffic scenarios, Yang Rening et al. [15] proposed improvements to YOLOv5s by substituting the upsampling module with the CARAFE operator, thereby broadening the target receptive field. They also replaced the loss function with EIoU, incorporated a small target detection layer, and modified the decoupling head to improve detection performance for small targets. Additionally, channel pruning techniques were employed to reduce the model size. These modifications facilitate effective monitoring of both small and occluded targets. The model shows promise in enhancing detection accuracy while simultaneously reducing its overall size. However, there is still considerable room for further improvement in practical deployment scenarios. In scenarios involving air doors, detection personnel typically utilize manual or infrared methods. Conversely, the machine vision-based monitoring algorithm for underground personnel has emerged as the predominant detection approach for individuals operating in subterranean environments. Li Xianguo et al. [16] proposed a detection algorithm for underground personnel using the Single Shot MultiBox Detector (SSD) network. By incorporating dense connection networks and residual networks, they improved both detection accuracy and real-time monitoring speed. The detection speed achieved was 48 Frames Per Second; however, the recognition speed remained relatively slow. Furthermore, the problem of missed detections is common in situations characterized by a high density of pedestrians or substantial occlusion. Zou Sheng et al. [17] introduced an enhanced CornerNet-Squeeze method for detecting personnel in coal mines, effectively addressing the challenges posed by complex underground environments and the limited detailed features at the edges of personnel targets. They implemented OctConv to strengthen feature extraction capabilities and employed a dual-scale image fusion algorithm to improve image quality. This approach facilitates increased detection accuracy while preserving the original model’s detection speed. The computational complexity of the model presents challenges for practical deployment, and the detection accuracy for large and medium-sized targets is somewhat diminished compared to the original model. The research conducted by Moawiah Alhulayil et al. [18] on relay networks demonstrates that the model’s performance can be significantly enhanced through the application of various advanced algorithms in complex environments. This finding provides a theoretical foundation for utilizing the improved model to enhance target detection capabilities in mine-related scenarios. Unlike vehicle and personnel detection, identification in air door scenarios requires the detection of both large and small targets. Although previous research has made advancements in various aspects, it has not achieved an optimal balance among lightweight design, detection accuracy, and detection speed. With the advancement of intelligent construction in coal mines, the detection of personnel and vehicles within mine safety monitoring systems has garnered increasing attention. The mining environment presents considerable challenges for target detection due to low illumination and complex backgrounds. Although current target detection methods have made significant progress, notable shortcomings remain in research pertaining to complex environments. These challenges include multi-target detection, difficulties in identifying small targets, and low visibility conditions specific to mine air door scenarios.

In light of this, the author has enhanced the YOLOv8n model and introduced a lightweight CGSW-YOLO model specifically designed for multi-target monitoring in air door scenarios. The primary contributions of this paper are as follows: The FasterNet network has been integrated into the C2f module of the backbone network, resulting in the design of the C2F-FASTER module. This innovation enables the model to maintain a lightweight configuration without sacrificing accuracy. To minimize redundant information generated during feature extraction, GhostConv has been introduced as a substitute for specific standard convolutions within the backbone network. The slim neck module has been designed by integrating GSConv and the cross-stage partial network VOV-GSCSP module into the neck architecture, effectively reducing both computational load and parameter count while preserving accuracy. Additionally, we have replaced the loss function with WIoUv3 to enhance the quality of boundary frames, significantly improving the model’s ability to locate and detect multiple targets in air door scenarios. This approach effectively meets the deployment requirements for video surveillance in coal mines, particularly concerning lightweight design and real-time performance. It aims to provide an efficient monitoring solution for man-vehicle interactions in intelligent coal mine air door scenarios.

## 2. Method and Principle

### 2.1. YOLOv8 Model

At present, object detection algorithms based on deep learning are developing rapidly. These algorithms can be categorized into two-stage and single-stage object detection methods according to their operational flow. Classical two-stage object detection algorithms include Fast R-CNN [19] and Faster R-CNN [20], among others. While these methods achieve high detection accuracy, they often suffer from prolonged processing times, making them challenging to implement in practical scenarios. In contrast, classic single-stage object detection algorithms such as SSD, YOLO, and RetinaNet integrate the tasks of localization and classification. As a result, they significantly enhance real-time detection performance compared to their two-stage counterparts.

The YOLOv8 model, which is developed based on the YOLOv5 architecture, comprises five distinct variants: YOLOv8l, YOLOv8m, YOLOv8s, YOLOv8n, and YOLOv8x. YOLOv8 builds upon the CSP gradient SHVDC concept introduced in YOLOv5 and incorporates the ELAN framework from YOLOv7. It replaces the C3 module with the C2f module while retaining the SPPF module. This design choice enables YOLOv8 to maintain a rich gradient flow while ensuring a lightweight architecture. The PAN and FPN architectures continue to be utilized within the neck network; however, the upsampled 1 × 1 convolutional module has been eliminated. In the detection head component, the coupling head has been replaced with a decoupling head, thereby achieving a separation between the classification and detection heads. Additionally, an anchor-free approach is adopted, eliminating the need for anchor frames.

### 2.2. CGSW-YOLOv8n Network Structure

**Figure 1:**
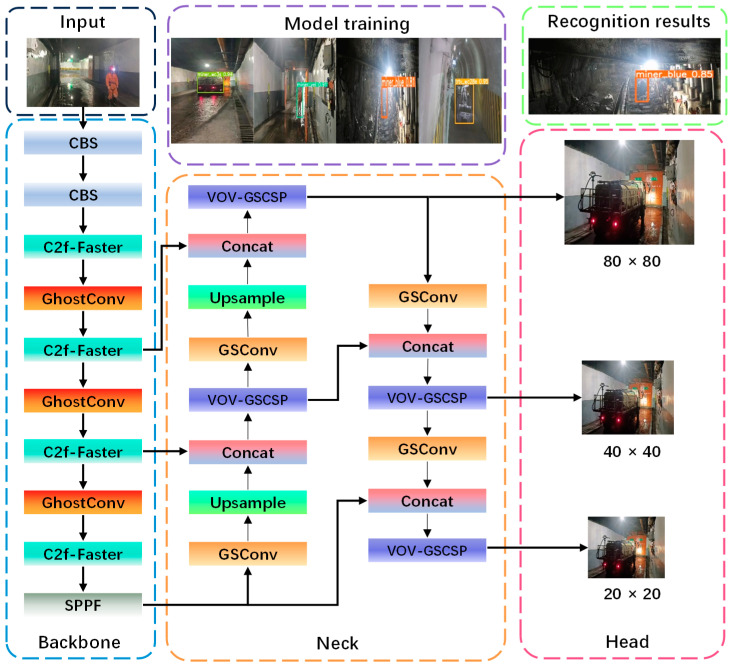
CGSW-YOLOv8n network structure.

In response to the challenges associated with detecting size variations in targets, managing excessive model size, and achieving real-time performance in coal mine air door scenarios, this paper introduces the CGSW-YOLO model, which utilizes YOLOv8n as the benchmark. Firstly, the lightweight FasterNet network architecture is integrated into the C2f module of the backbone network. This integration not only reduces computational complexity while preserving accuracy but also enhances the C2f model’s ability to extract multi-scale features. Secondly, a segment of the standard convolution in the backbone network has been restructured as GhostConv. This modification allows for the generation of additional feature maps through low-cost operations, thereby facilitating efficient feature extraction of targets in air door scenarios. Furthermore, a Slim-neck module has been reintroduced to enhance the speed of multi-scale feature fusion for various targets within these air door contexts. Finally, the loss function was modified to WIoUv3 to reduce the missed detection rate associated with low-quality images. The improved network architecture is depicted in Figure 1. In Figure 1, C2f-Faster is the feature extraction module. GhostConv is a lightweight convolutional module. A Slim-neck module as a feature fusion includes two parts, GSConv and VOV-GSCSP.

#### 2.2.1. C2f-Faster Model

In the process of multi-target recognition in mining, retaining redundant information during feature extraction is common due to the diversity of target objects and their similar sizes and shapes. Traditional lightweight networks typically utilize Depthwise Separable Convolution (DWConv) [21] or Group Convolution (GConv) for feature extraction demands (MAC), which can lead to a significant decline in detection accuracy. Chen et al. [22] further proposed a lightweight network called FasterNet, which is based on the concept of partial convolution.

**Figure 2:**
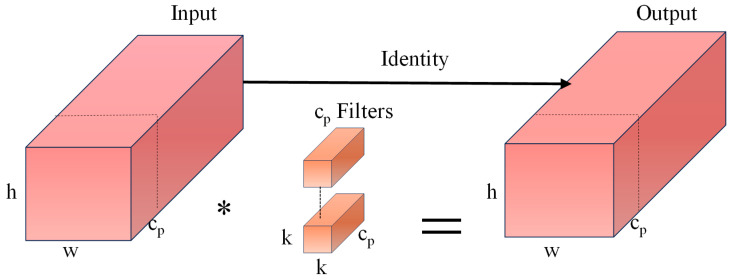
PConv structure.

In PConv, the convolution operation for extracting spatial features is conducted exclusively on one-fourth of the input channels, while the remaining channels remain unaltered. These unaltered channels are then concatenated with the processed one-fourth of the channels during the output stage. The structural diagram is illustrated in Figure 2. In Figure 2, * represents the convolution operation, *h* and *w* represent the height and width of the input feature map, respectively; *k* denotes the size of the convolution kernel; *c_p_* indicates the number of channels involved in the convolution process; *r* refers to the convolution rate; and *c* signifies the number of input channels. Therefore, by maintaining a consistent number of channels, both computational requirements and memory access are minimized. The computational method employed in *F_PConv_* is outlined in Equation (1), while the memory access technique, known as *M_MAC_*, is described in Equation (2):

(1) $$F_{PConv}=h\times w\times k^{2}\times c_{p}^{2}$$

(2) $$M_{MAC}=h\times w\times 2c_{p}+k^{2}\times c_{p}^{2}\approx h\times w\times 2c_{p}$$

(3) $$r=\frac{c_{p}}{c}$$

It can be observed from the aforementioned formula that the computational complexity of PConv is only one-sixteenth that of standard convolution, while the memory access requirements are approximately one-quarter of those associated with conventional convolution.

**Figure 3:**
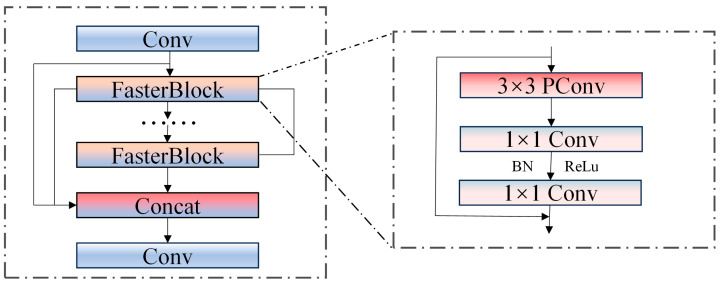
C2f-Faster structure.

The C2f module integrates standard convolution and series bottleneck modules, which complicates the network architecture when extracting features of personnel and vehicles. To address the issue of feature redundancy resulting from the extensive use of standard convolutions in feature fusion, we replace the bottleneck module within the C2f module with the Faster Block module designed in FastNet. Consequently, a new C2F-FASTER module has been developed. After the convolution operation, the input feature graph is directed into a two-branch channel. One of these branches utilizes the Faster Block module to process the feature graph. During this process, the feature graph undergoes one 3 × 3 PConv and two 1 × 1 convolutions, with batch normalization (BN) and ReLU activation applied after each 1 × 1 convolution. The other branch does not perform any operations. Ultimately, the feature maps produced by the two branches are concatenated. Following this, the combined feature maps undergo an additional convolution operation to yield the final feature maps. The structural diagram of C2f-Faster is illustrated in Figure 3.

The enhanced C2f-Faster module utilizes a multi-scale feature fusion mechanism to enhance the model’s robustness in detecting vehicles and personnel of varying target sizes under mine damper conditions. This approach allows the model to more effectively extract local feature information and process detections of targets across different scales. When identifying vehicles with substantial size disparities, the model’s recognition performance is significantly improved.

In the coal mine environment, image details are frequently obscured due to blurriness caused by variations in illumination or interference from dust. C2f-faster enhances the model through multi-scale feature fusion, which enables the extraction of effective features from images captured at different scales and under varying lighting conditions. This approach significantly improves the model’s robustness. Ye Yongjing et al. [23] introduced FasterNet as the backbone network to enhance the model’s capability for multi-scale feature processing and to improve its ability to locate license plates in environments characterized by strong light, reflections, and dust. The C2f-faster module employs a similar multi-scale fusion mechanism that enables the model to effectively tackle the extreme lighting and dust conditions prevalent in coal mine environments.

#### 2.2.2. GhostConv Module

The YOLOv8 backbone network utilizes stacked standard convolution and C2f modules for feature extraction, resulting in slow inference speeds and high computational costs on devices with limited memory and processing capabilities. To mitigate this issue, the author proposes replacing standard convolution in the backbone network with the lightweight GhostConv [24].

**Figure 4:**
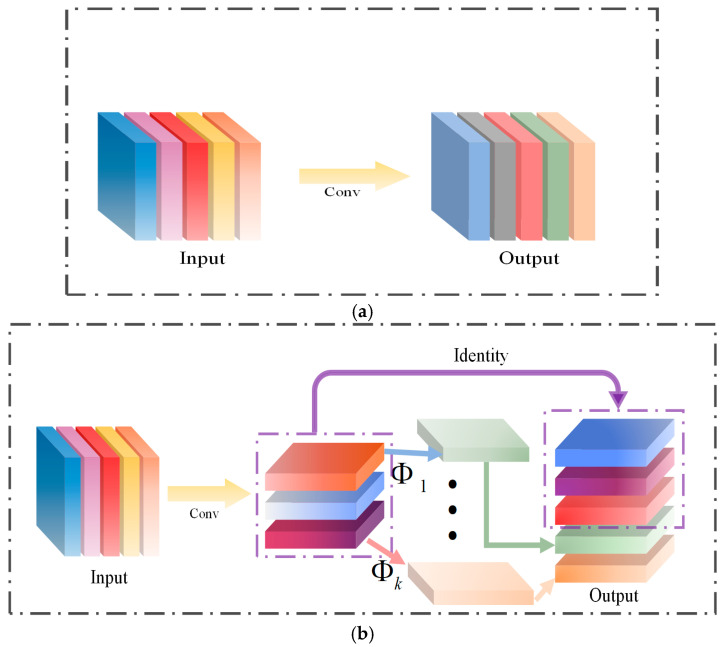
Standard convolution and GhostConv structures. (**a**) Standard convolution; (**b**) GhostConv.

Compared to standard convolution, which generates all feature maps simultaneously, GhostConv employs a two-step process. Initially, it utilizes a 1 × 1 convolution to reduce the number of channels in the feature maps. Subsequently, it leverages the feature maps produced in the previous step to perform inexpensive operations that generate new feature maps. Finally, the outputs from these two components are combined to yield the final feature map, as illustrated in Figure 4, which compares the structures of standard convolution and GhostConv.

For standard convolution, let the input feature be represented as $X\in R^{c_{1}\times h_{1}\times w_{1}}$, and the convolution kernel be denoted as $f\in R^{c_{1}\times k_{1}\times h_{1}\times n}$. The computational process for generating *n* feature maps is outlined in Equation (4):

(4) Y=X∗f+b

In the formula, *c*_1_ denotes the number of input channels, and *h*_1_ and *w*_1_ represent the height and width of the output feature map, respectively. Additionally, *k*_1_ signifies the size of the convolution kernel *f*, while n indicates the number of such kernels. The term *b* refers to the bias coefficient. The expression for the standard convolution computation, denoted as *F_DSC_*, is presented in Equation (5):

(5) $$F_{DSC}=c_{1}\times h_{1}\times w_{1}\times k_{1}\times k_{1}\times n$$

For GhostConv, the initial step involves generating *m* feature graphs $Y^{\prime }\in R^{h_{1}\times w_{1}\times m}$, through the application of 1 × 1 convolution. The computational process is illustrated in Equation (6):

(6) $$Y^{\prime }=X*f^{\prime }$$

The convolution kernel is represented as $f\in R^{c_{1}\times k_{1}\times k_{1}\times m}$, where *m ≤ n*, and the bias term *b* is disregarded. Subsequently, inexpensive linear operations are applied to all feature maps in *Y′* to produce *s* feature maps, with the calculation process detailed in Equation (7):

(7) $$y_{ij}=\varphi_{i,j}(y_{i}^{\prime }):\forall_{i}\in [1,m],j\in [1,s]$$

In the formula, *y_ij_* represents the feature graph generated in the second step, *φ_ij_* denotes the *j*-th feature graph produced by the 1 × 1 convolution operation in the first step, and $y_{i}^{\prime }$ indicates the *i*-th linear operation. Ultimately, the final output feature map is obtained through splicing, resulting in a total of *m* * *n* channels being generated. Therefore, the calculation of the *FLOPS* for the GhostConv process is presented in Equation (8).

(8) $$FLOPS=k_{1}\times k_{1}\times c_{1}\times \frac{n}{s}\times w_{1}^{\prime }\times h_{1}^{\prime }+\frac{n}{s}\times d\times d\times (s-1)\times w_{1}^{\prime }\times h_{1}^{\prime }$$

In the formula, *d* represents the convolution kernel of a linear operation, allowing for the determination of the number of parameters for both cases, as illustrated in Equation (9):

(9) $$p=\frac{k_{1}\times k_{1}\times c_{1}\times n\times w_{1}^{\prime }\times h_{1}^{\prime }}{k_{1}\times k_{1}\times c_{1}\times \frac{n}{s}\times w_{1}^{\prime }\times h_{1}^{\prime }+\frac{n}{s}\times d\times d\times (s-1)\times w_{1}^{\prime }\times h_{1}^{\prime }}\approx \frac{c\times s}{c+s-1}\approx s$$

It can be observed from the aforementioned formula that, compared to standard convolution, the computational complexity of GhostConv is approximately 1/s that of conventional convolution. Furthermore, it effectively eliminates redundant feature information without altering the number of channels in the output feature map. This characteristic contributes to a reduction in both model computation and overall model size.

Affected by low illumination and the specific equipment used in mining environments, vehicles and personnel are likely to be obscured when traversing areas with dampers. The GhostConv module enhances the model’s inference speed during actual deployment in these settings by generating a greater number of feature maps while minimizing computational requirements. This improvement strengthens the model’s ability to capture information related to low-level features, ensuring that object detection remains effective even under occlusion conditions [25].

Ye Yongjing et al. [26] introduced FasterNet as the backbone network to enhance the model’s capability for multi-scale feature processing and to improve its ability to locate license plates in environments characterized by strong light, reflections, and dust. The C2f-faster module utilizes a comparable multi-scale fusion mechanism that enables the model to effectively address the extreme lighting and dust conditions commonly found in coal mine environments. The model’s efficient computation and real-time processing capabilities allow it to swiftly process images in challenging environments while maintaining strong detection performance. Consequently, the integration of the GhostConv module enhances computational efficiency and improves real-time performance in complex settings. This optimization boosts the model’s performance under conditions of low illumination, dust interference, and target occlusion by refining its response speed, thereby further enhancing both the robustness and detection capabilities of the model.

#### 2.2.3. Slim-Neck Model

The YOLOv8 architecture integrates the C2f module within its Neck network; however, this approach has certain limitations in feature fusion. To address these issues, we propose replacing the standard convolution with GSConv and substituting the C2f module with the VOV-GSCSP module, thereby introducing a lightweight Slim-neck module [27].

Compared to standard convolution, depth-separable convolution involves a trade-off between detection accuracy and feature extraction capabilities. However, this method significantly reduces both the computational load and the number of parameters in the model. GSConv is a novel convolutional technique introduced by Li et al. [28], which combines standard convolution with depth-separable convolution by implementing a channel shuffle operation.

**Figure 5:**
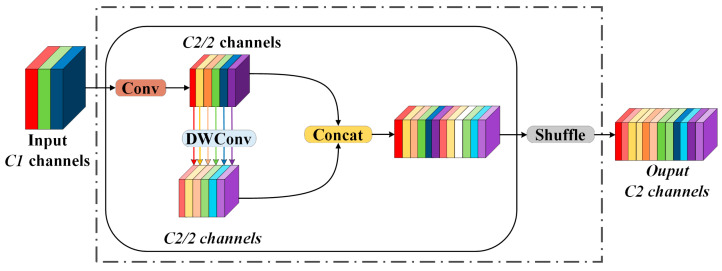
GSConv structure.

The GSConv module initially reduces the number of output channels in the feature graph to half of its original value through standard convolution. Subsequently, it applies depth-separable convolution operations to the resulting feature graph, ensuring that the output channel count remains unchanged at half. Finally, a channel mixing operation is performed to generate the final feature graph. The structure of this process is illustrated in Figure 5.

The formulas for calculating depth-separable convolution and GSConv are presented in Equations (10) and (11), respectively:

(10) $$F_{DWC\mathrm{onv}}=h_{2}\times w_{2}\times k_{2}\times k_{2}\times C_{out}$$

(11) $$F_{GSConv}=h_{2}\times w_{2}\times k_{2}\times k_{2}\times \frac{C_{out}}{2}(C_{in}+1)$$

In the formula, *h*_2_ and *w*_2_ represent the height and width of the output feature map, respectively. The variable *k*_2_ denotes the size of the convolution kernel, while *C_in_* and *C_out_* indicate the number of input channels and output channels, respectively.

**Figure 6:**
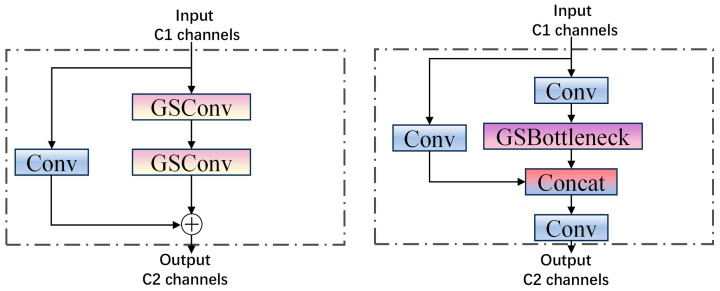
Structure of GSbottleneck and VOV-GSCSP.

According to the aforementioned formula, when the number of input channels is sufficiently large, the computational cost of GSConv is reduced to half that of standard convolution. Consequently, incorporating GSConv into the Neck network not only reduces model complexity but also enhances network accuracy while preserving image information. The VOV-GSCSP module is a critical bottleneck component developed from GSConv. It integrates the one-time aggregation module, VOVNet, with the interphase layout network. The structure of this module is depicted in Figure 6. The VOV-GSCSP module enhances the feature information of the receptive field by linking adjacent feature maps, thereby improving the efficiency of feature extraction for personnel and various types of vehicles. This approach not only simplifies the network model but also reduces the associated computational overhead.

The implementation of the Slim-neck module in image processing significantly enhances both the efficiency and accuracy of the model, particularly in the context of mine air door scenes. By minimizing redundant computations, this module greatly improves operational efficiency while ensuring precise detection of the device. Target occlusion, complex backgrounds, and variations in target size within mining environments often present significant challenges to target detection. However, the Slim-neck module not only enhances the model’s robustness in these intricate settings but also ensures detection accuracy and real-time performance through multi-scale feature fusion and structural optimization. The efficient design of this module further enables the model to meet real-time inspection requirements while maintaining a low computational burden, thereby improving the overall performance of the system.

In mining environments, the complexity of the background significantly increases the challenges associated with target detection. Factors such as low illumination and dust interference often lead to suboptimal performance of traditional target detection algorithms. The Slim-neck module enhances the model’s sensitivity to critical target features within images by streamlining redundant calculations. Even in intricate backgrounds, this model can maintain high-precision detection capabilities, thereby greatly improving its robustness. This optimization enables accurate target detection in conditions characterized by insufficient light or substantial interference.

In addition, targets within the mining environment often display dimensional variations due to factors such as distance and equipment positioning. The Slim-neck module, through its optimized design and multi-scale feature fusion, allows the model to maintain high detection accuracy when encountering targets of different sizes. This adaptability to changes in target dimensions ensures the stability of detection tasks. Zhou Libing et al. [29] introduced the Slim-neck module for pedestrian detection in unmanned vehicles, facilitating the efficient fusion of feature map information. This innovation significantly improved both the detection accuracy and speed of the model. The application of the Slim-neck module underscores its considerable value for target detection in complex environments.

#### 2.2.4. WIoU Loss Function

The loss function used in the target detection algorithm consists of both a bounding box regression loss function and a classification loss function. The bounding box regression loss function is designed to predict the bounding box through a learning process, ensuring that the location of the detected target closely aligns with the actual marked boundaries. The YOLOv8 model employs CIoU as the loss function for bounding box regression, with its structure detailed in Equations (12)–(14).

(12) $$CIOU=1-IOU+\frac{(x-x_{1}{)}^{2}+(y-y_{1}{)}^{2}}{w_{3}^{2}+h_{3}^{2}}+\alpha \nu$$

(13) $$\alpha =\frac{\nu }{L_{IOU}+\nu }$$

(14) $$\nu =\frac{4}{\pi^{2}}{\left(\tan^{-1}\frac{w}{h}-\tan^{-1}\frac{w_{3}}{h_{3}}\right)}^{2}$$

In the formula, *IOU* represents the Intersection over Union ratio between the predicted bounding box and the ground truth bounding box. The variables *x* and *y* denote the coordinates of the center point of the predicted box, while *x*_1_ and *y*_1_ indicate the coordinates of the center point of the ground truth box. The parameter *α* is a hyperparameter that influences model performance. Additionally, *w*_3_ and *h*_3_ refer to the maximum width and height of the overlapping region between the predicted box and the ground truth box.

**Figure 7:**
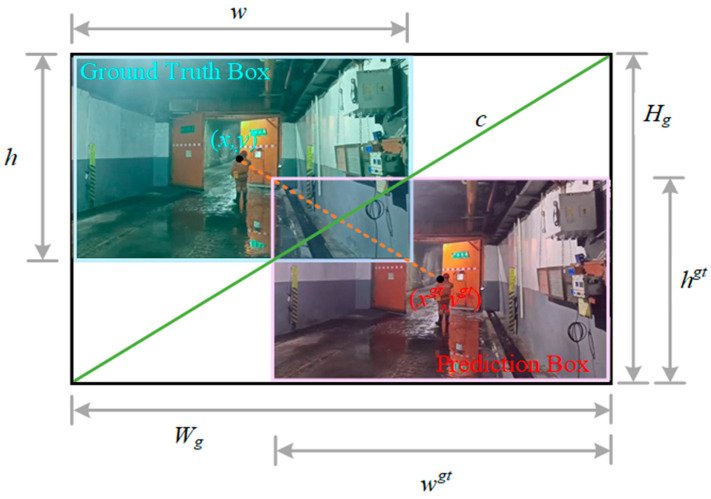
WIoU structure.

Considering the significant variations in the dimensions of real boundary boxes marked by different types of vehicle targets and the various tasks related to the identification of people and vehicles, as well as the inconsistent quality of samples, it is evident that higher-quality samples will improve the model’s fitting capability. Furthermore, the CIoU loss function imposes stringent requirements on the aspect ratio of geometries, and training on low-quality samples may adversely affect the model’s generalization performance. To address these issues, the WIoUv3 [30] loss function is proposed as a replacement for the CIoU loss function within the enhanced algorithm. The WIoU loss function, based on Focal EIoUv1, incorporates a dynamic non-monotonic focusing mechanism designed to evaluate the quality of anchor boxes by utilizing outliers instead of IoU. The expressions for WIoUv1 are presented in Equations (15) and (16), while the structural diagram is illustrated in Figure 7.

(15) $$L_{WIOUv1}=R_{WIOU}L_{IOU}$$

(16) $$R_{WIOUv1}=\exp\left(\frac{(x-x_{1}{)}^{2}+{\left(y-y_{1}\right)}^{2}}{w_{3}^{2}+h_{3}^{2}}\right)$$

The expression for WIoUv3 is presented in Equations (17)–(19).

(17) $$L_{WIOUv3}=rR_{WIOUv1}L_{IOU}$$

(18) $$r=\frac{\beta }{\delta \cdot \alpha^{\beta -\delta }}$$

(19) $$\beta =L_{IOU}^{*}/\bar{L_{IOU}}\in \left[0,+\infty \right)$$

In the formula, *R_WIOU_* represents the distance loss. The term *R_WIOUv_*_1_ ∈ [1,e) can enhance the anchor frame *L_IOU_* of average quality, while *L_IOU_* ∈ [0,1] has the potential to decrease the *R_WIOUv_*_1_ associated with high quality. Here, *α* and *δ* are hyperparameters, and *β* denotes an outlier. For samples that exhibit a significant degree of outlier characteristics, such as vehicles or personnel that are partially obscured or blurred, assigning a small gradient gain can substantially mitigate the influence of these low-quality samples on the training process. In our empirical experiments, we found that setting *α* = 2 and *β* = 3 yields the optimal gradient gain. $L_{IOU}^{*}$ indicates that $\bar{L_{IOU}}$ is decoupled from the computation graph, where $\bar{L_{IOU}}$ represents the dynamic average IoU value with momentum m. The dynamic updating of the IoU results in a corresponding adjustment in the mass distribution of the anchor frame. Consequently, WIoUv3 can implement a more rational gradient gain strategy during training, thereby enhancing overall detection performance and efficiency.

The WIoU module demonstrates an enhanced capability to capture features in unoccluded areas and improves the detection of edge details when the model is partially occluded through weighted calculations. In scenarios involving challenges such as low illumination, dust interference, and target occlusion, WIoU effectively optimizes the regression of the target frame, thereby facilitating improved extraction of edge information related to the target. At the same time, the fuzzy target can be weighted to reduce the information loss caused by its interference, thereby maintaining a high level of detection accuracy. Zhu Qin et al. [31] introduced the WIoU module to enhance the model’s positioning capabilities and significantly improve detection accuracy. This highlights the effectiveness and advantages of this module in complex environments.

## 3. Experiment and Analysis

### 3.1. Experimental Environment Configuration

The computer hardware used in this experiment is configured with a Windows 10 operating system, an Intel^®^ Core™ i5-12400F CPU (Intel, Santa Clara, CA, USA) operating at 2.5 GHz, an RTX 4060 Ti graphics card (NVIDIA, Santa Clara, CA, USA), and 32 GB of RAM. The software environment includes Python version 3.8.0, along with PyTorch version 1.8.1 and CUDA version 11.1.

The model parameters are configured as follows: the input image size is set to 640 × 640 pixels; the training cycle consists of 300 iterations; the batch size is established at 8; the initial learning rate is specified as 0.01; the momentum parameter is defined as 0.937; the weight decay factor is set to 0.0005; and early stopping criteria are applied with a threshold of 50 rounds without improvement.

### 3.2. Experimental Dataset

Due to the presence of numerous vehicles and individuals traversing the mine air door, it is essential to enhance the model’s generalization capabilities. To achieve this, it is necessary to gather comprehensive information on all vehicles and individuals that may pass through the air door during the data collection process. The image dataset is composed of various scenes of wind doors captured on-site at the Shanxi Jinshen Ciyao Coal Industry in Xinzhou, China, as well as from surveillance videos recorded at the Jinneng Holding Tashan Coal Industry in Datong, China. A total of 861 effective images depicting explosion-proof diesel trackless rubber wheels and personnel movement across various scenarios—including air doors, adits, and rubber conveyor heads—were collected through frame extraction from video surveillance and recordings. The dataset was annotated using the LabelImg software (version number is 1.8.6) and categorized into the following eight categories.

**Figure 8:**
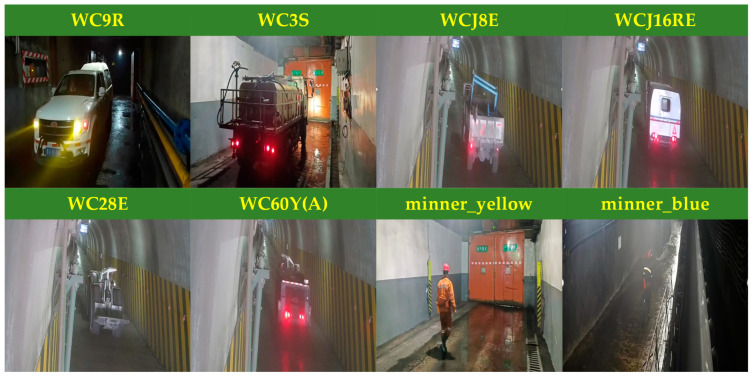
Data set samples of different vehicle types and jobs.

Due to the variety of vehicles operating in different scenarios, along with the various categories of miners navigating the coal mine, it is essential to improve safety measures for both personnel and company property in air door situations. Furthermore, there is a pressing need to enhance safety monitoring and inspection efforts within the coal mine. The various types of vehicles and personnel operating through the air door are categorized into eight distinct groups: WC9R explosion-proof trackless rubber-wheeled vehicles (WC9R), WC3S explosion-proof trackless rubber-wheeled vehicles (WC3S), WCJ8E explosion-proof trackless rubber-wheeled vehicles (WCJ8E), WC28E explosion-proof trackless rubber-wheeled vehicles (WC28E), WC60Y(A) explosion-proof trackless rubber-wheeled vehicles (WC60Y(A)), maintenance workers (miner_yellow), and coal workers (miner_blue). Samples of the different vehicle types and job categories are presented in Figure 8.

To mitigate the risk of overfitting during the training process and enhance the model’s performance, the dataset was augmented to include 2400 images using various data augmentation techniques. These techniques included flipping, translation, contrast enhancement, adaptive histogram equalization, and random occlusion. Subsequently, the dataset was partitioned into a training set, validation set, and test set in a ratio of 7:2:1.

### 3.3. Model Evaluation Indicators

In order to objectively assess the multi-objective intelligent recognition method for coal mine damper scenes, this experiment utilizes several performance evaluation metrics: Precision (P), Recall (R), Average Precision (AP), mean Average Precision (mAP), Parameters, and Frames Per Second (FPS).

Params is the number of model training parameters, the larger the number of parameters of the model, the heavier the computational burden during training and reasoning. FPS refers to the number of images that the model can process per second. The formulas for calculating the remaining indicators are presented in Equations (20)–(23).

(20) $$Prescision=\frac{TP}{TP+FP}$$

(21) $$Recall=\frac{TP}{TP+FN}$$

(22) $$AP=\int_{0}^{1}P\left(R\right)dR$$

(23) $$mAP=\frac{\sum_{1}^{N}AP_{i}}{N}$$

In the formula, *TP* represents the number of samples that have been correctly identified, while *FP* denotes the number of samples that have been incorrectly identified. *AP* refers to the area under the Precision–Recall (P–R) curve, and n indicates the total number of categories for the identified samples. Based on the dataset utilized in this study, *n* is equal to 8.

### 3.4. Ablation Experiment

To assess the effectiveness of the enhanced methods presented in this paper, YOLOv8n was utilized as the benchmark model for conducting ablation experiments. The results are displayed in Table 1 below. First, the C2f model of the backbone network is replaced with the C2F-faster model to improve feature fusion. Second, standard convolution in the backbone network is substituted with ghost convolution. Subsequently, the lightweight Slim-neck model is introduced for multi-scale feature fusion. Finally, the loss function is modified to WIoUv3.

As illustrated in Table 1, the incorporation of the C2f-Faster module into Model ① resulted in a 2.7% increase in Precision (P) and a 1.3% increase in mean Average Precision at IoU = 0.5 (mAP@0.5) compared to the original model. Additionally, there was a 12.3% reduction in the number of parameters. These findings suggest that integrating the C2f-Faster module into the backbone network significantly enhances the effectiveness of feature fusion. Model ② employs a lightweight ghost convolution approach. While maintaining a mean Average Precision (mAP) of 0.5, it achieves a 2.6% increase in Precision (P). Additionally, the number of parameters decreases by 6.3%, and the Frames Per Second (FPS) improves from 100 to 102.04.

These results indicate that the model successfully reduces its weight while preserving detection accuracy. In Model ③, the Slim-neck model is introduced independently, resulting in a 2.9% increase in Recall (R). The number of parameters is reduced by 7.0%, and the Frames Per Second (FPS) increased by 2.04%. Meanwhile, the mean Average Precision at an Intersection over Union (IoU) threshold of 0.5 (mAP@0.5) shows an improvement of 1.1%. This indicates that a slight compromise in detection speed is made to achieve enhanced accuracy. After substituting the loss function in Model ④, the metrics Precision (P), Recall (R), and mean Average Precision at an Intersection over Union (IoU) threshold of 0.5 (mAP@0.5) showed increases of 0.4%, 2.6%, and 0.3%, respectively, while the Frames Per Second (FPS) decreased by 28.6%. This indicates a significant improvement in model performance. Model ⑤, which integrates C2f-Faster and incorporates ghost convolution, demonstrated enhancements in Precision (P), Recall (R), and mean Average Precision at an IoU threshold of 0.5 (mAP@0.5) of 1.0%, 0.3%, and 0.1%, respectively. Simultaneously, the number of parameters decreased by 18.3%, while the FPS increased by 19%. This indicates that the model achieved a lightweight design while enhancing both detection accuracy and processing speed. The model ⑥ builds upon the foundation established by model ⑤ by incorporating the Slim-neck model. This integration has resulted in consistent improvements in Precision (P), Recall (R), and mean Average Precision at an Intersection over Union (IoU) threshold of 0.5 (mAP@0.5), while also reducing the number of parameters. These findings indicate that the introduction of the Slim-neck model not only enhances detection accuracy but also significantly improves detection speed. Model ⑦ represents the final enhanced version presented in this paper. Additionally, C2f-Faster, GhostConv, Slim-neck, and WIoUv3 have been integrated into the framework. The performance of this model has yielded optimal results, with Precision (P), Recall (R), mean Average Precision at an IoU threshold of 0.5 (mAP@0.5), and Frames Per Second (FPS) reaching 88.4%, 93.9%, 98.0%, and 135.14 Frames Per Second, respectively.

### 3.5. Contrast Experiment

#### 3.5.1. Comparative Analysis of Various Models

To further validate the superiority of the CGSW-YOLO model, we conducted comparative experiments with several current mainstream algorithms, including YOLOv3-Tiny, YOLOv5s, YOLOv7-Tiny, YOLOv8s, YOLOv8n, Faster-RCNN, EfficientDet [32], RE-DETE [33], and Yolov11n [34]. Under consistent experimental parameters and identical software and hardware environments, we compared various metrics, such as Precision (P), Recall (R), mean Average Precision (mAP), the number of parameters, Frames Per Second (FPS), floating point operations per second (FLOPS), and model size. The results are presented in Table 2 below.

Table 2 illustrates that the Precision (P) of the YOLOv3-Tiny model surpasses that of the CGSW-YOLO model by 4.7%. When compared to YOLOv5s, YOLOv7-Tiny, YOLOv8s, and YOLOv8n, the mean Average Precision (mAP) is greater by 1.1%, 6.6%, 1.8%, and 1.3%, respectively. However, the mean Average Precision (mAP) of the YOLOv3-Tiny model is slightly lower than that of the CGSW-YOLO model. However, the parameters, floating point operations per second (FLOPS), and model size of the Yolov5s model are less favorable compared to the improved algorithm, which presents challenges for the practical deployment of equipment in underground coal mines. The Precision (P) of the Yolov5s model is 4.0% higher than that of the CGSW-YOLO model. However, its mean Average Precision (mAP), parameters, and Frames Per Second (FPS) are lower than those of the CGSW-YOLO model by 1.5% and 48.1%, respectively. Additionally, the parameters, floating point operations per second (FLOPS), and model size of the Yolov5s model are all greater than those of the improved algorithm. The mean Average Precision (mAP) of the CGSW-YOLO model was found to be 7.0%, 2.2%, and 1.7% higher than that of the YOLOv7-Tiny, YOLOv8s, and YOLOv8n models, respectively. The parameters of the CGSW-YOLO model demonstrated reductions of 66.7%, 78.7%, and 21.6%, respectively. Additionally, the floating point operations per second (FLOPS) for the CGSW-YOLO model decreased by 53.0%, 78.2%, and 23.5%. Furthermore, the Frames Per Second (FPS) performance of the CGSW-YOLO model improved by 64.5%, 40.5%, and 26.0%. Lastly, the model size of the CGSW-YOLOv8n variant was reduced by 67.5%, 77.9%, and 20.6%, respectively.

As illustrated in Table 2, the mean Average Precision (mAP) of the Faster R-CNN model and the RE-DETE model is only 0.4% and 0.6% lower than that of the CGSW-YOLO model, respectively. However, it is noteworthy that both models exhibit significantly higher numbers of parameters, computational demands, and overall model sizes compared to the CGSW-YOLO model. At the same time, the Frames Per Second (FPS) of these two models decreased by 65.7% and 64.3%, respectively, compared to the CGSW-YOLO model. Although both models demonstrate high accuracy in detection performance, they do not sufficiently meet the lightweight requirements necessary for practical deployment. The mean Average Precision (mAP) of the EfficientDet model and the YOLOv11n model was found to be 1.8% and 2.4% lower, respectively, than that of the CGSW-YOLO model. Furthermore, their reductions in Frames Per Second (FPS) were 28.9% and 29.4%, respectively, compared to the CGSW-YOLO model. At the same time, the EfficientDet model demonstrated a 21.6% increase in floating point operations per second (FLOPS) compared to the CGSW-YOLO model. However, it also contained a significantly larger number of parameters than the CGSW-YOLO model, which impeded its ability to maintain Frames Per Second (FPS) while ensuring high detection accuracy. Compared to the benchmark model, YOLOv8n, the YOLOv11n model has a smaller size. However, this reduction compromises both detection accuracy and real-time performance, as it sacrifices parameters and floating point operations per second (FLOPS). In practical applications within mining environments, it is clear that using YOLOv8n as the benchmark model offers a more effective balance between model accuracy and Frames Per Second (FPS) capabilities.

Therefore, the comparative experiments presented above demonstrate that the CGSW-YOLO model effectively balances lightweight design with high precision. This further emphasizes the advantages of the improved method proposed in this paper.

#### 3.5.2. Different Convolution Contrast Experiments

To evaluate the effectiveness of the GhostConv module, we utilized the reference model YOLOv8n to replace the ODConv [35], DWConv, and CondConv [36] modules, while ensuring consistent experimental conditions for comparative analysis. The results of these experiments are presented in Table 3 below.

As shown in Table 3, the accuracy of all convolution types has improved compared to the YOLOv8n model. Among the various convolution methods, GhostConv demonstrates the highest mean Average Precision (mAP), achieving an impressive 97.4%. Although ODConv exhibits a lower average accuracy than GhostConv, it surpasses it by 2.0% in Frames Per Second (FPS). CondConv can achieve a maximum performance of 232.56 FPS, albeit at the expense of mAP. DWConv results in a modest reduction of 0.4% in mAP and a decrease of 2.0% in FPS, while simultaneously maintaining minimal parameters, floating point operations per second (FLOPS), and model size. After integrating the YOLOv8n model with GhostConv, ODConv, CondConv, and DWConv, the performance metrics of GhostConv show significant improvements. Specifically, Precision (P), mean Average Precision (mAP), and Frames Per Second (FPS) increased by 2.6%, 1.1%, and 2.0%, respectively, compared to the original model. Furthermore, the number of parameters, floating point operations per second (FLOPS), and model size were reduced by 6.3%, 2.5%, and 6.3%, respectively, when compared to the baseline model. To summarize, GhostConv not only reduces the number of parameters and floating point operations per second (FLOPS) but also significantly enhances FPS during the feature extraction stage. This approach demonstrates clear and comprehensive advantages, effectively meeting the monitoring requirements in the context of mine air door scenarios.

#### 3.5.3. Comparison of Different Loss Functions

To evaluate the effectiveness of the WIoU loss function, comparative experiments were conducted using the GIoU, EIoU, and SIoU loss functions. The results of these experiments are presented in Table 4 below.

As illustrated in Table 4, the WIoU loss function employed in this study demonstrates a significant improvement over the CIoU loss function utilized by the benchmark model. Specifically, it enhances the mean Average Precision (mAP) by 0.3%, increases Precision (P) by 0.4%, boosts Recall (R) by 2.6%, and elevates Frames Per Second (FPS) performance by 31.58%.

The mean Average Precision (mAP) of the Weighted Intersection over Union (WIoU) is slightly lower than that of the Generalized Intersection over Union (GIoU) and the Enhanced Intersection over Union (EIoU) loss functions, with differences of 0.1% and 0.4%, respectively. However, the Frames Per Second (FPS) performance of WIoU exceeds that of the other three loss functions. This suggests that WIoU is more suitable for real-time monitoring on mobile devices.

#### 3.5.4. Visualization Experiment

**Figure 9:**
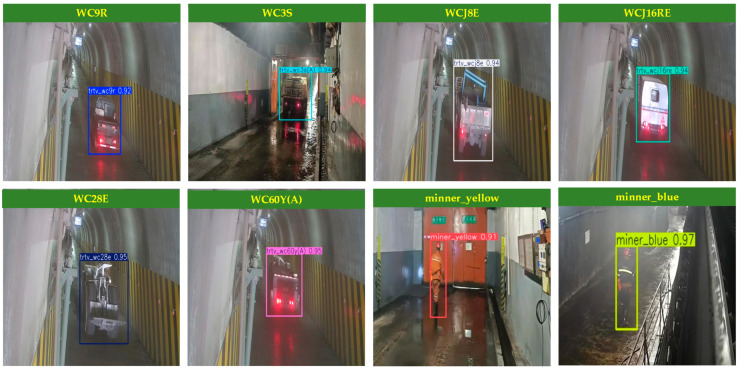
All category detection images of the CGSW-YOLOv8n model.

**Figure 10:**
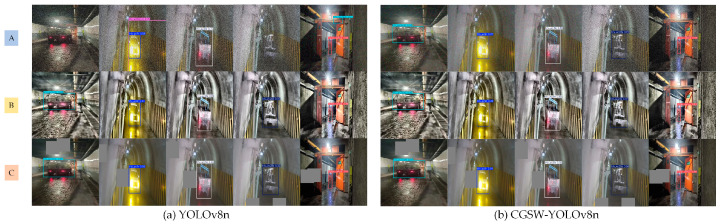
Comparison of detection effects in actual scenes.

The visualization results of the final improved model across all categories of the dataset are presented in Figure 9. To effectively illustrate the detection performance of the enhanced algorithm, a visual comparison was conducted between the YOLOv8n model and the CGSW-YOLO model, with the corresponding results displayed in Figure 10. 

In Figure 10, scene A represents noise, scene B illustrates occlusion, and scene C depicts contrast enhancement. The figure presents the detection results for various categories of vehicles and personnel.

As illustrated in Figure 10a, both models are capable of performing the detection task; however, the YOLOv8n model exhibits instances of missed detections and false positives during the detection process. For example, it fails to detect WC3S and WC28E vehicles. Additionally, there are cases of misidentification where WC9R vehicles are incorrectly classified as WC60Y(A) vehicles, as well as erroneous detections involving miner_yellow personnel, among others. The detection performance of the YOLOv8n model is suboptimal under noisy conditions. Furthermore, in scenarios involving occlusion and contrast enhancement, the detection efficacy remains inadequate.

As illustrated in Figure 9 and Figure 10, the CGSW-YOLO model effectively detected various vehicles and personnel, with no instances of missed or incorrect detections. Furthermore, the detection confidence under conditions of noise, occlusion, and contrast enhancement showed a slight improvement compared to that of the YOLOv8n model. Therefore, the CGSW-YOLO model is more proficient at accurately detecting vehicles and individuals in mine air door scenarios.

## 4. Discussion

The CGSW-YOLO model proposed in this study demonstrates an effective capability to identify personnel and vehicles within mine air door scenarios, achieving high detection accuracy and speed while maintaining a lightweight design. Despite advancements in several areas, this study acknowledges certain limitations, including the following:

(1) The model’s generalization capability across various mining environments remains a concern. Although datasets from two coal mines were used for training and testing, they do not comprehensively represent the diverse geological conditions and variations in equipment layout encountered in practice. Consequently, the model’s adaptability to different real-world environments requires further enhancement. Future research should focus on expanding the dataset by incorporating additional coal mine data that reflects a broader range of geological characteristics and equipment configurations, thereby improving the model’s robustness and generalizability;
(2) Although the proposed model demonstrates significant improvements in detection speed and efficiency, there is still potential for further optimization under extreme conditions, such as low illumination, severe occlusion, and excessive coal dust interference. Future research could explore more advanced data enhancement techniques, refine the model architecture, and improve its robustness to effectively address these challenges;
(3) In future research, we will continue to optimize the model to improve its detection capabilities across various industrial scenarios. This includes applications such as defect monitoring in manufacturing and navigation for autonomous vehicles.

## 5. Conclusions

(1) In this paper, we propose a multi-object monitoring model, CGSW-YOLO, which is based on an enhanced version of YOLOv8n specifically designed for coal mine air door scenarios. First, the FasterNet module is integrated into the C2f module of the backbone network. Next, GhostConv is utilized to replace a portion of the standard convolution operations within the backbone network, thereby reducing the computational load and accelerating model detection. Subsequently, we introduce a Slim-neck module composed of GSConv and VOV-GSCSP to improve the feature fusion capabilities of the neck network. Finally, we replace the loss function with WIoUv3 to mitigate the impact of low-quality anchor frames on model performance.
(2) The experimental results indicate that the CGSW-YOLO model effectively reduces both the number of model parameters and floating point operations per second (FLOPS), leading to an increased recognition speed. Furthermore, it exhibits a lower incidence of missed detections while maintaining high accuracy. These characteristics align with the monitoring requirements for air door scenarios in coal mines. In future research and development, the Frames Per Second (FPS) and model size will be further optimized to facilitate the subsequent deployment of the model on mobile devices.

## Figures and Tables

**Table 1:** Ablation experiment table.

Model	C2f-Faster	GhostConv	Slim-Neck	WIoU	P%	R%	mAP@50%	Parameters /M	FPS /f·s^−1^
Yolov8n					88.2	92.4	96.3	3.01	100.00
①	√				90.9	90.4	97.6	2.64	100.00
②		√			90.8	92.4	97.4	2.82	102.04
③			√		87.4	95.3	97.2	2.80	71.42
④				√	88.6	95.0	96.6	3.01	110.88
⑤	√	√			91.2	92.7	97.4	2.46	119.00
⑥	√	√	√		90.7	93.0	97.6	2.36	131.58
⑦	√	√	√	√	88.4	93.9	98.0	2.36	135.14

Note: √ indicates the introduction of this module.

**Table 2:** Comparison experiment table of different models.

Model	P%	R%	mAP@0.5	Parameters/M	FLOPS/G	FPS/f∙s^−1^	Size/MB
Yolov3-tiny	92.9	90.1	97.6	8.69	12.9	72.36	17.5
Yolov5s	92.2	93.1	96.5	7.03	15.8	70.05	14.5
Yolov7-tiny	87.8	87.5	91.0	6.00	13.2	48.04	12.3
Yolov8s	92.9	93.1	95.8	11.10	28.4	80.42	22.6
Yolov8n	88.2	92.4	96.3	3.01	8.1	100.00	6.3
Faster R-CNN	90.9	90.4	97.6	137.10	226.4	46.30	108.0
RE-DETE	90.8	92.4	97.4	42.78	130.5	48.25	86.0
EfficientDet	90.1	93.4	96.2	3.90	5.1	96.15	16.2
Yolov11n	90.1	91.6	95.6	2.58	6.3	95.47	5.5
CGSW-YOLO	88.2	93.9	98.0	2.36	6.2	135.14	5.0

**Table 3:** Different convolution contrast experiment table.

Model	P%	R%	mAP@0.5	Parameters/M	FLOPS/G	FPS/f∙s^−1^	Size/MB
Yolov8n	88.2	92.4	96.3	3.01	8.1	200.00	6.3
ODConv	91.6	91.2	97.3	3.01	8.2	208.33	6.3
CondConv	91.1	89.6	95.0	3.02	7.8	232.56	6.3
DWConv	91.4	92.5	95.9	2.64	7.6	196.08	5.6
GhostConv	90.8	92.4	97.4	2.82	7.9	204.08	5.9

**Table 4:** Different loss function contrast experiment table.

Model	P%	R%	mAP@0.5	FPS/f∙s^−1^
CIoU (Yolov8n)	88.2	92.4	96.3	100
GIoU	89.8	93.5	96.5	116.28
EIoU	88.5	92.8	97.0	121.95
SIoU	90.1	93.4	96.2	119.05
WIoU	88.6	95.0	96.6	131.58

## Data Availability

All data generated or analyzed during this study are included in this published article. The original contributions presented in the study are included in the article, further inquiries can be directed to the corresponding author.
